# Histone deacetylase inhibition in the treatment of acute myeloid leukemia: the effects of valproic acid on leukemic cells, and the clinical and experimental evidence for combining valproic acid with other antileukemic agents

**DOI:** 10.1186/1868-7083-5-12

**Published:** 2013-07-30

**Authors:** Hanne Fredly, Bjørn Tore Gjertsen, Øystein Bruserud

**Affiliations:** 1 Section for Hematology, Institute of Medicine, University of Bergen, N-5021, Bergen, Norway; 2 Department of Medicine, Haukeland University Hospital, Jonas Lies 65, 5021, Bergen, Norway

**Keywords:** Acute myeloid leukemia, Older patients, Disease stabilization, Valproic acid

## Abstract

Several new therapeutic strategies are now considered for acute myeloid leukemia (AML) patients unfit for intensive chemotherapy, including modulation of protein lysine acetylation through inhibition of histone deacetylases (HDACs). These enzymes alter the acetylation of several proteins, including histones and transcription factors, as well as several other proteins directly involved in the regulation of cell proliferation, differentiation and apoptosis. Valproic acid (VPA) is a HDAC inhibitor that has been investigated in several clinical AML studies, usually in combination with all-*trans* retinoic acid (ATRA) for treatment of patients unfit for intensive chemotherapy, for example older patients, and many of these patients have relapsed or primary resistant leukemia. The toxicity of VPA in these patients is low and complete hematological remission lasting for several months has been reported for a few patients (<5% of included patients), but increased peripheral blood platelet counts are seen for 30 to 40% of patients and may last for up to 1 to 2 years. We review the biological effects of VPA on human AML cells, the results from clinical studies of VPA in the treatment of AML and the evidence for combining VPA with new targeted therapy. However, it should be emphasized that VPA has not been investigated in randomized clinical studies. Despite this lack of randomized studies, we conclude that disease-stabilizing treatment including VPA should be considered especially in unfit patients, because the possibility of improving normal blood values has been documented in several studies and the risk of clinically relevant toxicity is minimal.

## Introduction

Acute myeloid leukemia (AML) is caused by clonal expansion of myeloblasts that have lost the normal regulation of differentiation and proliferation; this causes bone marrow accumulation of the leukemic cells, and thereby general bone marrow failure, and eventually leukemization and organ infiltration [1]. The median age at the time of diagnosis is 65 to 70 years. Although the prognosis of AML has improved during the last decade, this is mainly true for younger adults who can receive the most intensive treatment, including stem cell transplantation, whereas the overall survival (OS) for older AML patients remains poor [2].

The conventional intensive chemotherapy for AML usually has a treatment-related mortality of approximately 5% [3]. The early mortality for patients receiving allogeneic stem cell transplantation is approximately 20 to 25%, even for patients without severe comorbidity [4]. Thus, a large group of patients will be unfit for the most intensive therapeutic strategies due to age [5,6], severe non-hematological diseases, reflected in their comorbidity score [7], or poor performance status [6,8]. Another group of older patients should not receive intensive chemotherapy with the intention of remission induction because they have high-risk disease and remission is unlikely [6,7,9]. In these patients, less intensive treatment based on histone deacetylase (HDAC) inhibition may be an alternative, either alone or in combination with other low-toxic strategies [10].

### Valproic acid (VPA) in combination with all-*trans* retinoic acid (ATRA)

#### Biological and clinical effects of VPA

Valproic acid (VPA) is a short-chain fatty acid that is an established antiepileptic agent, with proven activity also in bipolar disorder, migraine and neuropathic pain [11]. It is generally well tolerated, but use of VPA in early pregnancy is associated with an increased risk of congenital malformations, including spina bifida in 1 to 2% of cases, and the malformations seem to be related to the drug’s anti-tumor properties [12].

VPA functions as a powerful HDAC inhibitor [13]. Acetylation is one of the main histone modifications leading to the opening of chromatin and promoting gene transcription, whereas histone deacetylation promotes chromatin condensation and represses gene transcription [14]. HDACs are overexpressed in malignant tissue, including leukemic blasts [15]. HDAC inhibitors may therefore result in re-expression of silenced tumor suppressor genes in cancer cells, and potentially lead to cellular differentiation and apoptosis [14].

VPA has a wide range of effects on AML cells and the results from previous studies are summarized in Table 1[16-38]. These observations clearly demonstrate that VPA can induce differentiation, and has antiproliferative and proapoptotic effects in AML cells. However, patients are most likely heterogeneous with regard to both susceptibility to VPA and molecular mechanisms mediating the antileukemic effects. Direct effects on the leukemic cells seem most important, but indirect effects mediated through increased antileukemic immune reactivity may also contribute. Few studies have explored VPA as monotherapy in AML [39,40] and only low response rates have been seen (3 to 5%).

**Table 1:** Functional or molecular targeting effects of valproic acid (VPA) on acute myeloid leukemia (AML) cells

**Intracellular signaling and trafficking**	
	Expression of the CXCR-4 receptor is decreased in CD34- AML cells, whereas increased expression is observed in CD34+ leukemic cells [25]. *In vivo* treatment with VPA in combination with ATRA alters the phosphorylation status and the phosphoresponsiveness of several intracellular signaling pathways, but the effects differ between patients [35]. VPA increases p21 but downregulates c-Myc expression at a transcriptional level [18]. Modulation of CBP activity and interaction with PML nuclear bodies may contribute to the effects of VPA [38]. The nucleolar morphology and function is altered [36]. VPA also alters the overall expression pattern of the various HDACs [16].
**AML cell proliferation, differentiation and apoptosis**	
	VPA has an antiproliferative effect that is dose-dependent. The effects differ between patients and at lower concentrations even enhancement of proliferation is seen for a subset of patients [37]. VPA reprograms the differentiation program in AML cells, especially in cells with a myelomonocytic phenotype [19]. Animal studies of APL suggest that terminal granulocytic differentiation can also be seen [30]. Differentiation is especially seen in t(8;21) AML cells [31].
**Effects on leukemic stem cells**	
	In animal models of APL, VPA causes rapid disease regression in induction of granulocytic differentiation, but discontinuation is associated with immediate disease relapse, suggesting that leukemia-initiating cell activity is not affected by VPA [30]. Studies in human cells also suggest that VPA spare or increase immature AML cells during *in vitro* culture [17]. Direct associations between epigenetic modifications and reprogramming of normal as well as cancer stem cells are now emerging for other malignancies [33].
**Effects on t(8;21) AML**	
	In contrast to other AML subsets, VPA inhibits not only the mature AML cells but also the immature progenitors in AML1/ETO [17]. The drug targets the AML1/ETO-HDAC complex, and thereby alters gene expression and induces differentiation [31]. VPA has specific effects in this AML subset. The drug induces differentiation followed by apoptosis and accompanied by increased expression of repressed AML1 target genes [31].
**Effects on antileukemic immune reactivity**	
	In combination with 5-AZA, VPA causes induction of specific T cell responses against cancer-associated antigens [24]. The drug also increases the susceptibility to NK cell-mediated lysis through upregulation of NK cell ligands on the leukemic cells [32,34]; the NK cells then target leukemic stem cells [29]. This effect is also seen for ATRA [34]. AML cells are also sensitized to TRAIL/Apo2L-induced apoptosis by VPA [27]. Spontaneous *in vitro* apoptosis is associated with immunogenic apoptosis with HSP release and calreticulin exposure; VPA does not interfere with this expression during stress-induced apoptosis [20].
**Chemosensitivity and chemoresistance**	
	A recent experimental study suggested that VPA induces a broad chemoresistance phenotype in AML cells [26]. However, clinical data does not support this observation since VPA can be combined with cytarabine, hydroxyurea and 6-mercaptopurin in the treatment of AML patients [22,23]. One marker of sensitivity may be UTX (KDM6A), which has a functional relationship between protein acetylation and lysine-specific methylation [21]. Resistance programs have also been identified that compensate for the HDAC inhibitor-induced global hyperacetylation, and these programs include MAPKAPK2, HSP90AA1, HSP90AB1 and ACTB [21]. One study also suggested serum HSP90 as a possible marker of sensitivity to VPA [22]. Cellular high expression of FOSB may be another sensitivity marker [28].

5-AZA, 5-azacytidine; AML, acute myeloid leukemia; APL, acute promyelocytic leukemia; ATRA, all-*trans* retinoic acid; CXCR-4, C-X-C chemokine receptor type 4; FOSB, FBJ murine osteosarcoma viral oncogene homolog B; HDAC, histone deacetylase; HSP, heat shock protein; HSP90, heat shock protein 90; HSP90AA1, heat shock protein 90 kDa alpha (cytosolic), class A member 1; HSP90AB1, heat shock protein 90 kDa alpha (cytosolic), class B member 1; MAPKAPK2, mitogen-activated protein kinase-activated protein kinase 2; NK, natural killer; PML, promyelocytic leukemia; UTX, ubiquitously transcribed tetratricopeptide repeat, X chromosome; VPA, valproic acid.

### Biological and clinical effects of ATRA combined with VPA

In 1981, all-*trans* retinoid acid (ATRA) was proven to differentiate human acute promyelocytic leukemia (APL) cells *in vitro*[41], and ATRA has now changed APL from a highly fatal to a highly curable disease [42-44]. In APL, the absence of ATRA leads to HDAC activities, inducing chromatin condensation and transcriptional repression [45]. Pharmacological levels of ATRA then induce a conformational change in the promyelocytic leukemia (PML)/retinoic acid receptor α (RARα) fusion oncoprotein, thereby allowing the release of HDAC complexes and recruitment of transcriptional co-activators with growth suppression and differentiation induction [46].

Several clinical studies have investigated the combination of ATRA and VPA in non-APL variants of AML. This combination has antileukemic activity in experimental *in vitro* studies [46] and is usually well tolerated by patients. The results of these clinical studies are summarized in Table 2[19,39,40,47-51]. Based on these results, the following conclusions can be made: 1) the combination of VPA and ATRA has clinically relevant antileukemic activity; 2) the combination is well tolerated, and the most common side effects are dose-dependent and reversible fatigue, and gastrointestinal toxicity; 3) the antileukemic activity can be seen at serum levels below the generally accepted therapeutic levels used for antiepileptic treatment; 4) the treatment is also safe for older patients and can be combined with low-toxicity chemotherapy; 5) a clinical effect is only observed for a minority of patients, and in several studies this is only observed in 20 to 40% of the included patients; and 6) the most common effect on peripheral blood cells is increased platelet counts, whereas complete hematological remission is very uncommon.

**Table 2:** **Valproic acid (VPA) and all-*trans *retinoic acid (ATRA) in the treatment of acute myeloid leukemia (AML): summary of clinical studies**

**Study**	**Number of patients**	**Median age (range) (years)**	**Diagnosis**	**Treatment**	**Results**	**Common toxicity**
Ryningen *et al*., 2009 [51]	24	71 (47 to 86)	AML	ATRA 22.5 mg/m^2^ × 2 day 1 to 15. VPA and theophylline iv day 3 to 7, thereafter, orally indefinitely. Serum levels of theophylline 50 to 100 μM, VPA 200 to 700 μM.	MDS criteria: 9/22 patients had increasing cell counts and 4/22 patients (18%) HI [52,53]. Median survival 64 days (7 to 644 days).	Two patients had atrial fibrillation. Fatigue and nausea were most common.
Bellos *et al*., 2008 [47]	22	71.5 (41 to 89)	AML (95%) or MDS	VPA 150 to 300 mg/d. ATRA 45 mg/m^2^/d for 14 days.	MDS criteria: four patients HI-P and one patient HI-E. Treatment duration 37 days (4 to 730 days).	Usually well tolerated. Two patients had ATRA syndrome and two patients had continuous fever.
Cimino *et al*., 2006 [19]	8	61.5 (31 to 69)	AML (88%) or CML blast crisis	VPA 15 to 30 mg/kg/d with serum levels 50 to 110 μg/ml. ATRA 45 mg/m^2^ from day 14. Cytoreductive drugs if hyperleukocytosis.	Two patients (25%) HI and five patients had stable disease. No clinical response according to AML criteria [54]. Survival 119 days (60 to 184 days).	One patient had grade III hepatic toxicity, and one patient had vertigo and tremor.
Kuendgen *et al*., 2006 [40]	58	71 (42 to 86)	AML	VPA reaching serum levels 50 to 100 μg/ml. ATRA either 80 mg/m^2^ days 1 to 7 every second week, or ATRA 15 mg/m^2^/d from day 4. Total of 31 patients received VPA monotherapy. Cytoreductive drugs if hyperleukocytosis.	AML criteria: one patient CR, one patient CRi and one patient PR; 5% response. MDS criteria: 16% responses, 34% stable disease and 50% progressive disease. No difference between treatment groups. Median OS 6.74 months.	Seven patients had tremors. Four patients had grade I/II skin toxicity, three patients had grade I/II gastrointestinal toxicity and one patient had pleural effusion.
Bug *et al*., 2005 [48]	26	69 (59 to 84)	AML (92%) or advanced MDS	VPA 5 to 10 mg/kg/d, escalating doses to 5 to 64 mg/kg. ATRA 45 mg/m^2^/d. Cytoreductive drugs if hyperleukocytosis.	One patient PR, one patient had minor response (2/19) and no patients CR; 10% responses. Survival not reported.	Three patients had grade IV neurological or pulmonary toxicity and there were 21 events with grade III toxicity.
Raffoux *et al*., 2005 [50]	11	82 (70 to 85)	AML	VPA reaching serum levels 50 to 100 μg/ml. ATRA 45 mg/m^2^/d from day 7. Theophylline reaching serum levels 10 to 15 μg/ml. Cytoreductive drugs if hyperleukocytosis.	AML criteria: one patient CR and two patients CRi. According to MDS criteria: two additional patients with HI. Survival 6 months (1 to 28 months).	Main side effects were tremor, mental confusion and theophylline-related palpitations.
Kuendgen *et al.*, 2005 [39]	75	67 (21 to 84)	AML (43%) or MDS	VPA reaching serum concentrations 50 to 100 μg/ml. Total of 66 patients received VPA monotherapy. ATRA 80 mg/m^2^ days 1 to 7 every second week.	MDS criteria: 18 patients responded (24%), one patient CR, one patient PR, 16 patients HI and 25 patients had stable disease. Median response duration 4 months (2 to 27 months).	Skin and gastrointestinal toxicity.
Pilatrino *et al*., 2005 [49]	20	70 (63 to 80)	AML (65%) or MDS	VPA 10 mg/kg/d escalating to 311 to 693 μM. ATRA 45 mg/m^2^/d. Cytoreductive drugs if hyperleukocytosis.	MDS criteria: 30% patients HI and no patients CR. Median duration of response 189 days (63 to 550 days).	Neurologic toxicity and bone pain.

AML, acute myeloid leukemia; ATRA, all-*trans* retinoic acid; CML, chronic myeloid leukemia; CR, complete remission; CRi, complete remission incomplete (peripheral blood criteria not fulfilled); HI, hematological improvement; HI-E, hematological improvement in erythrocytes; HI-P, hematological improvement in platelet counts; MDS, myelodysplastic syndrome; OS, overall survival; PR, partial remission; VPA valproic acid.

ATRA therapy has dramatically improved the prognosis of APL [42] and has also been used in the treatment of non-APL AML [39,40,48,51]. ATRA toxicity, as reported during treatment of APL, includes the ATRA or APL differentiation syndrome [55]. This syndrome has an incidence of 2 to 27% and a mortality rate of 2 to 15% [55,56]. Other frequent side effects include dryness of mucosa, headache, and increased transaminases and triglycerides [55]. Serious side effects of ATRA 45 mg/m^2^/d are very uncommon when VPA plus ATRA are used in the treatment of non-APL AML (Table 2) [47].

### VPA in combination with demethylating agents

#### The clinical experience of demethylating agents as monotherapy in human acute myeloid leukemia (AML)

The nucleoside analogues decitabine and 5-azacitidine (5-AZA) are the two demethylating agents most extensively studied. Decitabine inhibits DNA methyltransferase (DNA MTase) and causes DNA hypomethylation. It has shown convincing activity in myelodysplastic syndrome (MDS) in a large, phase III European trial, including 233 patients aged 60 years or older [57]; 13% of patients receiving decitabine achieved complete remission (CR) and 6% achieved partial remission (PR), compared with none in the best supportive care group. In AML, a phase II multicentre study, including 55 patients older than 60 years, 25% of patients achieved CR and 29% of patients had stable disease [58].

5-AZA is a ribose structure, which needs to be metabolized by ribonucleotide reductase to be incorporated into DNA; it cannot be combined with hydroxyurea due to pharmacodynamic interactions [59]. 5-AZA has been approved for the treatment of MDS and has demonstrated increased survival, compared to conventional treatment in a randomized phase III study, including 358 patients [60]. The median OS was 24.5 versus 15 months, and after 2 years 50.8% of patients in the treatment group were alive versus 26.2% in the conventional treatment group. Similarly, the same clinical study showed a superior 2-year survival rate for the subgroup of patients with bone marrow blast counts between 20 to 30% (that is, patients with AML according to current World Health Organization (WHO) criteria), with 2-year survival rates of 50 and 16% in the treatment and conventional group, respectively, and fewer days in hospital for the 5-AZA treatment group [61]. Based on its documented safety and efficiency, 5-AZA is an acceptable alternative for treatment of AML, at least in selected subsets of older patients.

### Biological and clinical effects of combining VPA and demethylating agents

The epigenetic changes in AML include altered DNA methylation and histone acetylation leading to gene silencing. Several clinical studies have investigated the effect of combining VPA with DNA demethylating agents [62]. The results from five different studies are summarized in Table 3[63-67]. Additional biological studies of these patients demonstrated that DNA hypomethylation and histone H3/H4 acetylation can be induced by this treatment [66]. Taken together, these studies suggest that the toxicity of this regimen is acceptable even in older patients; encephalopathy/confusion has been described in a relatively high number of patients in certain studies, but this would be an expected dose-dependent effect of VPA. The studies of VPA plus ATRA in older patients suggests that it may be difficult to reach the ordinary therapeutic serum levels of VPA in these patients; however, relatively low serum levels may be sufficient for induction of an antileukemic response [51]. Taken together, these studies suggest that VPA plus a demethylating agent is more effective than combining VPA and ATRA. The results summarized in Tables 2 and 3 demonstrate that the OS and CR rates are generally better for patients treated with VPA plus demethylating agent. However, with the lack of randomized clinical studies it is not possible to make a firm conclusion about the preferred regimen combination.

**Table 3:** Combination of histone deacetylase (HDAC) inhibition and demethylating agents: summary of clinical studies

**Study**	**Number of patients**	**Median age (years)**	**Diagnosis**	**Treatment**	**Results**	**Common toxicity**
Raffoux *et al*., 2010 [64]	65	72	AML (85%) or high-risk MDS	5-AZA 75 mg/m^2^. VPA 35 to 50 mg/kg po day 1 to 7. ATRA 45 mg/m^2^ po day 8 to 28. Six cycles.	After six cycles, 34 patients survived: 13 patients (38%) CR, two patients (6%) PR and 14 patients (41%) had stable disease. Median OS was 12.4 months.	Confusion 33 events, infection 76 events.
Blum *et al*., 2007 [63]	25	70	AML	Decitabine 15 to 20 mg/m^2^/d iv days 1 to 10 every 28 days. VPA 15 to 20 mg/kg days 5 to 21 in ten patients.	Response rate was 44%: four patients CR, four patients CRi and three patients PR (AML criteria). Survival not reported.	Neutropenic fever (64%), fatigue and infection (both 48%) were most common.
Soriano *et al*., 2007 [65]	53	69	AML (92%) or high-risk MDS	5-AZA 75 mg/m^2^/d. VPA 50 to 75 mg/kg/d days 1 to 7. ATRA 45 mg/m^2^ days 3 to 5. Treatment repeated every 3 weeks.	Overall response rate was 42%: 12 patients (22%) CR. Survival not reported.	Two events of grade IV and 11 events of grade III non-hematological toxicity; mainly fatigue or other neurotoxicities.
Garcia-Manero *et al*., 2006 [66]	54	60	AML (89%) or MDS	Decitabine 15 mg/m^2^/d iv. VPA 20 to 50 mg/kg days 1 to 10. Treatment repeated every fourth week.	Twelve patients (22%) had responses: 10 patients CR and two patients CRp. OS 6 months (0.6 to 20.2 months).	Fatigue, nausea and diarrhoea were the most common non-hematological toxicities.
Maslak *et al*., 2006 [67]	10	66.5	AML (80%) or MDS	5-AZA 75 mg/m^2^/d days 1 to 7. Sodium phenylbutyrate 200 mg/kg/d iv days 8 to 12. Treatment repeated every 21 to 28 days.	Three patients (30%) PR and two patients (20%) had stable disease. Duration of response was 45 days (37 to 136 days). Survival not reported.	Three patients had neutropenic fever. Nausea, dizziness and fatigue were common.

5-AZA, 5-azacytidine; AML, acute myeloid leukemia; ATRA, all-*trans* retinoic acid; CR, complete remission; CRi, complete remission incomplete (peripheral blood criteria not fulfilled); CRp, incomplete platelet recovery; MDS, myelodysplastic syndrome; OS, overall survival; PR, partial remission; VPA, valproic acid.

### VPA versus other histone deacetylase (HDAC) inhibitors in the treatment of human AML

Except for VPA, several other HDAC inhibitors have been developed, as described in a recent review article [14], and used in clinical studies [68,69]. In 2007, vorinostat was the first HDAC inhibitor to be approved as treatment for the malignant disease primary cutaneous T cell lymphoma. Phase I and phase II studies in AML have been undertaken combining vorinostat with demethylating agents or chemotherapy. The response rates have varied between 4 to 86% with combined treatment showing the best results [70-74]. Mocetinostat (MGCD0103) has shown response rates from 10 to 30% with best results when combined with 5-AZA [14]*.* Entinostat was also combined with 5-AZA and showed a response rate of 44% in 31 patients, including 7.5% CR, whereas a monotherapy phase I trial showed no responses [14]. Panobinostat (LBH-589) and romidepsin have also been developed, and they showed no response in phase I trials as monotherapy for different types of leukemia and MDS in 15 and 20 patients, respectively [14]. Other HDAC inhibitors have been developed but data are still preliminary. As for VPA, it is essential to also combine these HDAC inhibitors with other active substances to increase the response rates. Finally, butyrate is still considered as a therapeutic tool in clinical oncology [75], but, as previously reviewed, the results from available clinical studies in hematological malignancies are not promising [76,77].

To conclude, VPA is still the best investigated HDAC inhibitor for the treatment of human AML, both with regard to low-toxicity disease-stabilizing treatment and more intensive remission-inducing treatment.

### Low-toxicity treatment with cytotoxic drugs: an alternative to VPA or a possibility of combination therapy?

Several clinical reports have described the combined use of VPA with low-toxicity conventional chemotherapy. As described below, VPA has been combined with cytarabine, hydroxyurea and 6-mercaptopurine (6-MP); three drugs that interact with nucleic acid synthesis through targeting of different intracellular molecules.

### Low-dose cytarabine

Cytarabine is an analogue of deoxycytidine and shares the same metabolic pathway as this deoxycytidine [78]. Cytarabine is metabolized to its active triphosphate form, which inhibits the enzyme DNA polymerase alpha and is incorporated into elongating DNA strands, thereby causing chain termination. However, this triphosphate form is degraded by several enzymes and this intracellular metabolism is also regulated by several feedback mechanisms. The cytarabine-mediated cytotoxicity caused by its active triphosphate form is thereby determined by a complex interplay between cytarabine dose, anabolism, catabolism, and endogenous purine and pyrimidine levels [78].

Cytarabine is a cornerstone of AML treatment [79]. In 1987, treatment with low-dose subcutaneous cytarabine in 129 AML patients resulted in a CR rate of 31% and PR rate of 18% [80]. Later, low-dose cytarabine was combined with various therapeutic agents, including other cytotoxic drugs, growth factors, arsenic trioxide and homoharringtonine [81-84]. In these four trials, the CR rates were 14 to 52% and the cytarabine dose varied between 15 to 20 mg/m^2^/d for 10 to 14 days. Although low-dose cytarabine is frequently used, the mechanism of action is not known in detail and may include differentiation induction, as well as direct cytotoxic effects [85].

The biological effects and clinical results of low-dose cytarabine have been recently reviewed [85]. The following conclusions can be made based on the currently available clinical studies in human AML: 1) the treatment has an antileukemic effect and can improve survival; 2) the treatment is most effective for patients with low- and intermediate-risk disease, and the improved survival is mainly due to a beneficial effect in a minority of patients achieving complete hematological remission; 3) survival is not improved for patients with high-risk cytogenetic abnormalities; 4) the cytarabine dose used in these studies varies between 10 and 40 mg/m^2^ given once or twice daily, and the duration of treatment is usually 10 days but up to 21 days has been used; 5) treatment-related mortality is seen at least when using the higher doses, but this mortality shows a wide variation between studies; and 6) combination with other cytotoxic drugs is possible, but this has been investigated mainly in very small clinical studies and some of these studies suggest that the treatment-related mortality will then be increased.

Although effective, a major drawback of subcutaneous cytarabine is that patients often have to attend the outpatient department to receive the subcutaneous injections.

### Hydroxyurea

DNA synthesis requires production of deoxyribonucleotides and ribonucleotide reductase is necessary for this production [86]. Hydroxyurea inactivates the enzyme directly through electron donation but also indirectly through conversion to nitric oxide. This enzyme is also necessary for DNA repair and causes a block at the G1/S transition of the cell cycle, and thereby has cytotoxic effects.

Hydroxyurea has been used for decades in the treatment of hematologic malignancies; its activity is based on inhibition of the ribonucleotide reductase enzyme, and thereby inhibition of DNA synthesis [87]. Despite the lack of supporting clinical data, hydroxyurea is often used in AML for older patients not eligible for intensive chemotherapy. In a retrospective analysis of 244 older patients, 52% of patients received hydroxyurea and no significant difference in survival compared to treatment with 6-thioguanine (6-TG) or low-dose cytarabine was found [88]. A recent randomized clinical trial showed that low-dose cytarabine was superior to hydroxyurea in 217 older patients [89], resulting in CR rates of 18% versus 1%, respectively. In this study, the cytarabine dose was standardized to 20 mg/m^2^ twice daily for 10 days every 4 to 6 weeks, whereas hydroxyurea was administered to keep the white blood cell count below 10 x 10^9^/l. The addition of ATRA made no significant difference. Another small study showed acceptable safety and a CR rate of 41.6% for high-dose hydroxyurea (100 mg/kg/d) administered daily until bone marrow aplasia, or for a maximum of 30 days for 12 patients with poor-risk AML [90].

### 6-mercaptopurine (6-MP)

6-MP is a thiopurine and an analog of hypoxanthine [91,92]. The mechanism for its cytotoxic activity seems to be intracellular conversion to 6-TG nucleotides and methylated derivatives, which have a cytotoxic effect. Several molecular mechanisms may contribute to this effect, including incorporation of 6-TG nucleotides into nucleic acids. The drug has been used in AML therapy, mainly in palliative or maintenance treatment [93,94]. In Japanese trials, the drug was used at the dose of 70 mg/m^2^ for 7 days in repetitive cycles. A major advantage of both hydroxyurea and 6-MP is the oral administration, which makes management of outpatients easier.

### The possibility to combine VPA with low-toxicity cytotoxic therapy

Clinical studies have shown that VPA, possibly together with ATRA, can be combined with low-dose cytarabine [20,95,96], hydroxyurea and 6-MP [23,95]. The results from the first three studies of VPA plus low-dose cytarabine are conflicting. One study concluded that the combination had limited clinical effect [96], while induction of CR was seen in the two other studies [20,95]. The largest study included 36 patients treated with continuous administration of VPA, intermittent oral ATRA (21.5 mg/m^2^ for 14 days every third month) and subcutaneous cytarabine (10 mg/m^2^ once daily for 10 days every third month) [95]. If cytarabine could not control hyperleukocytosis, it was replaced by hydroxyurea or 6-MP to maintain the peripheral blood blast count below 50 x 10^9^/l and to avoid symptoms of leukostasis [97]. In this study, the median age of the patients was 77 years (range 48 to 90 years), 11 patients responded to the treatment according to the MDS response criteria and two of these patients achieved complete hematological remission. The responders had a median survival of 171 days (range 102 to >574 days) and most of this time was spent outside hospital. These results suggest that a subset of patients will benefit from this treatment, and this is supported by a third study [20].

Experimental studies suggest that VPA may also be combined with other therapeutic agents in the treatment of human AML [98-109]. These results are summarized in Table 4.

**Table 4:** Possible combinations of valproic acid (VPA) with other therapeutic agents: current experimental evidence

**Agent**	**Evidence**	**Study**
Curcumin	Curcumin is a natural anticancer agent that affects the expression of NF-κB, Bcl-2 and Bax in leukemic cells. The combination with VPA causes upregulation of Bax with proliferation arrest, sub-G1 DNA accumulation and cell death in the HL-60 AML cell line. The effect is dependent on p38 activation.	Chen *et al.*, 2010 [100]
Folate receptor beta	The folate receptor beta mediates antiproliferative effects in AML cells and VPA upregulates the expression of this receptor. VPA and ATRA, combined with targeting of this receptor, may therefore have additive or synergistic antileukemic effects.	Qi and Ratnam, 2006 [105]
HSP90 inhibition	Co-treatment of the AML1/ETO-expressing Kasumi-1 cell line with VPA and the HSP90 inhibitor 17-AAG causes a synergistic inhibition of downstream signaling of mutated c-KIT.	Yu *et al.*, 2011 [108]
Hydralazine	Hydralazine is a nontoxic agent with DNA MTase-inhibiting effects. A clinical study suggested that the combination of hydralazine and VPA was a nontoxic treatment with an antileukemic effect *in vivo*. The effect has not been compared with VPA in combination with decitabine or 5-AZA.	Candelaria *et al.*, 2011 [99]
mTOR inhibition	Studies in AML cell lines show no additive proapoptotic effects, but only a limited number of cell lines were examined. However, in other experimental models of Flt3-ITD-transformed cells, VPA and mTOR inhibitors had synergistic proapoptotic effects.	Cai *et al.*, 2006 [98]; Ryningen *et al.*, 2012 [106]
NF-κB inhibition	Experimental studies suggest that the antileukemic effect of DNA MTase and HDAC inhibition is not only caused by epigenetic mechanisms, but also by additional and independent inhibition of NF-κB. Specific NF-κB inhibitors are now being developed and the antileukemic effects of proteasome inhibitors are also most likely caused by NF-κB inhibition.	Fabre *et al.*, 2008 [101]
p53 agonism, nutlin	The p53 agonist nutlin was combined with VPA, and the two drugs caused a synergistic induction of p53-dependent apoptosis in AML cell lines and primary AML cells. This synergism was also demonstrated in xenograft models of human AML.	McCormack *et al.*, 2012 [103]
Proteasome inhibitors, including bortezomib	This combination has an antiproliferative effect with cell cycle arrest of AML cell lines. Apoptosis is induced through caspase activation, and inhibition of cyclin D and telomerase is induced. The two drugs have synergistic effects. This synergism is also seen for other proteasome inhibitors, and, at least in certain experiments, the antileukemic effect is stronger for the proteasome inhibitors NPI-0051 and PR-171 than for bortezomib.	Fuchs *et al.*, 2009 [102]; Nie *et al.*, 2012 [104]; Wang *et al.*, 2011 [107]
sTRAIL	When VPA was combined with an anti-CD33 single chain fragment linked to sTRAIL, the two agents had synergistic effects on apoptosis induction in primary human AML cells.	ten Cate *et al.*, 2009 [109]

17-AAG, 17-*N*-allylamino-17-demethoxygeldanamycin; 5-AZA, 5-azacytidine; AML, acute myeloid leukemia; ATRA, all-*trans* retinoic acid; Bax, Bcl-2-associated X protein; Bcl-2, B-cell lymphoma 2; DNA MTase, DNA methyltransferase; HDAC, histone deacetylase; HSP90, heat shock protein 90; ITD, internal tandem duplications; mTOR, mammalian target of rapamycin; NF-κB, nuclear factor kappa-light-chain-enhancer of activated B cells; sTRAIL, soluble tumor necrosis factor-related apoptosis-inducing ligand; VPA, valproic acid.

### Best supportive care versus disease-stabilizing treatment based on VPA in unfit AML patients: should VPA be recommended even though randomized clinical trials are not available?

Best supportive care in AML generally refers to treatment with antibiotics and transfusions of blood products. Low-intensity therapy is often offered to control leukocytosis [110]. Retrospective analyses of a group of 244 AML patients not fit for standard treatment, but with 72.5% of patients receiving hydroxyurea, low-dose cytarabine or 6-TG, showed a median OS of 178 days (range 1 to 3,278) [88]. Eighty per cent of patients survived less than 12 months. Another retrospective study, including 2,657 AML patients older than 65 years, showed that 86% of patients died within 1 year [111]. Median OS was 2 months, ranging from 1 month for patients aged 85 years and older, to 3 months for patients aged 65 to 74 years. In this last study, 30% of patients received low-toxicity chemotherapy and the average age of these patients was 73 years, whereas the average age of the whole group was 77 years. Median OS for treated patients was 6 months longer than for the untreated patients, regardless of age at the time of diagnosis [111]. Similarly, a systematic review of clinical studies published between 1989 and 2006, including a total of 12,370 AML patients with median age 70 years, showed a median OS for patients receiving best supportive care of only 7.5 weeks [112]. Approximately 50% of these patients received intensive induction therapy, 30% received non-intensive chemotherapy and 20% received best supportive care.

These studies clearly demonstrate that unfit AML patients only receiving supportive care usually have a very short survival. Disease-stabilizing treatment based on VPA offers an opportunity of improved peripheral blood cell counts, to such a degree that the risk of bleeding or infections and the need for erythrocyte transfusions is reduced, compared with the pre-therapy situation. Is it then justified to recommend VPA-based treatment to these patients in the absence of randomized clinical studies, or should they still receive only supportive treatment? In our opinion, the answer is that VPA-based treatment should be recommended, because: 1) improved normal cell counts is an advantage that will reduce the risk of potentially severe complications; 2) all available studies suggest that this advantage can be achieved with acceptable toxicity; and 3) the low-toxicity justifies that this treatment is used for a period of several weeks even though the response rate is less than 50%.

### Future perspectives: the development of VPA as an antileukemic agent

#### Autologous antileukemic immune reactivity: experimental artefact or clinical reality?

The strategy to use tumor-specific vaccines as an individualized treatment was suggested several decades ago [113], and *in vivo* experiments in mice have supported this strategy [114]. However, whether enhancement of antileukemic immune reactivity is possible in humans (for example through vaccination with AML-associated antigens) requires further study. The use of such an approach as a low-toxicity strategy in older patients is supported by several observations. First, primary human AML cells can undergo immunogenic apoptosis with exposure of calreticulin, and release heat shock protein 70 (HSP70) and heat shock protein 90 (HSP90); this may initiate or enhance the development of antileukemic T cell reactivity [115]. Second, a prognostic impact of serum heat shock protein (HSP) levels has been suggested [116-119], possibly caused by soluble HSPs that facilitate immunogenic presentation of the client proteins. Finally, our recent study demonstrated that AML patients are heterogeneous with regard to systemic levels of immunoregulatory cytokines and patients could be subclassified based on their serum cytokine profiles; the effect of immunotherapy may therefore vary between patient subsets [22]. Thus, the possibility to enhance autologous antileukemic immune reactivity should be studied further as a low-toxicity treatment in human AML, and this strategy should combine with antileukemic chemotherapy [120]. Immunological side effects should be evaluated in future clinical studies of VPA treatment in human AML.

### What are the response criteria for studies of AML-stabilizing therapy?

The MDS response criteria developed by the International Working Group (IWG) for MDS [52,53] have been used to describe the effects of AML patients receiving disease-stabilizing treatment. The alternative IWG criteria for response in AML [54] are developed for AML patients treated with intensive chemotherapy, with the intention to induce complete hematological remission. However, with a palliative treatment strategy, minor hematological improvement or disease stabilization are also important in AML. Furthermore, the question of duration of the response is important in the MDS criteria. Duration of the response is certainly important for AML patients receiving disease-stabilizing therapy, but the durations are generally expected to be shorter in disease-stabilizing treatment of highly aggressive AML than for MDS patients with a much longer expected survival even without disease-directed therapy. Responses of shorter duration would therefore be of biological as well as clinical relevance in patients with AML. Complete hematological remission of any duration is important and may lead to longer survival even in patients receiving low-intensive treatment [54]. However, the MDS criteria are more detailed and provide the best opportunity to describe minor responses more accurately.

In a previous study exploring the combination of VPA, ATRA and theophylline in unfit AML patients, increased normal peripheral blood cell counts were also described for patients who did not fulfill the criteria for hematological improvement, as defined by the MDS criteria [51]. In this study, improvements of platelet levels, increased neutrophil or reticulocyte counts, or transfusion independency of durations shorter than 8 weeks, were reported. This is an alternative way of describing minor responses during palliative treatment in AML. Reporting of such small responses is justified because AML is usually a rapidly progressive disease and any spontaneous improvement is regarded as unexpected. However, the best solution would be to gain a general agreement on specific response criteria for patients receiving AML-stabilizing therapy.

### How should quality of life (QoL) be evaluated in early clinical trials?

Quality of life (QoL) is reduced in AML patients particularly at the time of diagnosis, due to the symptoms and signs of the disease, the information about the diagnosis and prognosis, and the initial treatment, but it usually improves and stabilizes [121,122]. There is no difference in QoL in older AML patients receiving intensive or non-intensive treatment when comparing the pre-treatment basic values, and the values after diagnosis and during treatment [123,124]. Different instruments and questionnaires are used to quantitatively measure QoL in AML, as well as in other hematologic malignancies, but the European Organization for Research and Treatment of Cancer (EORTC) QLQ-C30 questionnaire is one of the most commonly used scientific tools [121-123]. Evaluation of QoL is often not included in early phase II studies, but is important in larger randomized trials. An alternative for phase II studies is to report parameters, such as time in hospital versus days at home, to describe the situation of the patients, and such factors are most likely important for the patients’ QoL. Since previous studies have shown that QoL is related to response to therapy [125], it is likely that treatment-induced disease stabilization or even remission induction due to low-intensity treatment would lead to an improvement in QoL.

## Review and conclusion

Epigenetic strategies in AML are regarded as promising. The detection of reversible epigenetic changes reflected in the chromatin structure has increased our understanding of leukemia development and identified new therapeutic targets [126-128]. In addition to clinical trials with HDAC inhibitors, the number of trials with demethylating agents is also increasing and the combination of these two epigenetic strategies seem to have synergistic effects [129,130] (Table 3). HDAC inhibitor monotherapy has limited effects in AML and this treatment should be combined with other antileukemic agents in future clinical studies [39,40]. Particularly in the treatment of older AML patients, new targeted therapies should be tried and epigenetic strategies then represent well tolerated alternatives. Further increase of response rates can hopefully be made through development of low-toxicity combination therapy. Thus, in future, HDAC inhibitors should form part of the AML treatment, at least for older patients or patients unfit for intensive chemotherapy. A future role of VPA in the treatment of myeloproliferative diseases, including AML, is also supported by recent observations suggesting that this agent may be useful in chronic myeloproliferative neoplasms [131,132].

The available studies of VPA therapy in human AML have demonstrated that HDAC inhibition is a therapeutic strategy that should be investigated further. Future clinical studies should address the question of whether VPA, or any other drugs, should be the preferred HDAC inhibitor and investigate the optimal drug(s) to combine with HDAC inhibition. Randomized clinical trials are also needed to compare HDAC inhibition with alternative therapeutic approaches. HDAC inhibition as a therapeutic strategy should be considered, particularly in patients unfit for more intensive chemotherapy. This view is based on the available results from several clinical studies, which shows that VPA is the HDAC inhibitor most extensively investigated in human AML, this treatment can induce a clinically relevant improvement in peripheral blood cell counts and stabilization of the clinical status for a subset of AML patients, and the risk of clinically relevant toxicity is minimal**.**

## Abbreviations

17-AAG: 17-*N*-allylamino-17-demethoxygeldanamycin; 5-AZA: 5-azacytidine; 6-MP: 6-mercaptopurine; 6-TG: 6-thioguanine; AML: Acute myeloid leukemia; APL: Acute promyelocytic leukemia; ATRA: All-*trans* retinoic acid; Bax: Bcl-2-associated X protein; Bcl-2: B-cell lymphoma 2; CML: Chronic myeloid leukemia; CR: Complete remission; CRi: Complete remission incomplete (peripheral blood criteria not fulfilled); CRp: Incomplete platelet recovery; CXCR-4: C-X-C chemokine receptor type 4; DNA MTase: DNA methyltransferase; EORTC: European Organization for Research and Treatment of Cancer; FOSB: FBJ murine osteosarcoma viral oncogene homolog B; HDAC: Histone deacetylase; HI: Hematological improvement; HI-E: Hematological improvement in erythrocytes; HI-P: Hematological improvement in platelet counts; HSP: Heat shock protein; HSP70: Heat shock protein 70; HSP90: Heat shock protein 90; HSP90AA1: Heat shock protein 90 kDa alpha (cytosolic), class A member 1; HSP90AB1: Heat shock protein 90 kDa alpha (cytosolic), class B member 1; ITD: Internal tandem duplications; iv: Intravenous; IWG: International Working Group; LBH-589: Panobinostat; MAPKAPK2: Mitogen-activated protein kinase-activated protein kinase 2; MDS: Myelodysplastic syndrome; MGCD0103: Mocetinostat; mTOR: Mammalian target of rapamycin; NF-κB: Nuclear factor kappa-light-chain-enhancer of activated B cells; NK: Natural killer; OS: Overall survival; PML: Promyelocytic leukemia; po: Per os; PR: Partial remission; QoL: Quality of life; RARα: Retinoic acid receptor α; sTRAIL: Soluble tumor necrosis factor-related apoptosis-inducing ligand; UTX: Ubiquitously transcribed tetratricopeptide repeat, X chromosome; VPA: Valproic acid; WHO: World Health Organization.

## Competing interests

The authors declare that they have no competing interests.

## Authors’ contributions

HF, BTG and ØB contributed to the writing of the article. All authors read and approved the final manuscript.
